# Luminescent Down‐Conversion Semiconductor Quantum Dots and Aligned Quantum Rods for Liquid Crystal Displays

**DOI:** 10.1002/advs.201901345

**Published:** 2019-10-11

**Authors:** Abhishek K. Srivastava, Wanlong Zhang, Julian Schneider, Jonathan E. Halpert, Andrey L. Rogach

**Affiliations:** ^1^ State Key Laboratory of Advanced Displays and Optoelectronics Technologies Department of Electronic and Computer Engineering Hong Kong University of Science and Technology Hong Kong SAR China; ^2^ Department of Materials Science and Engineering, and Centre for Functional Photonics (CFP) City University of Hong Kong Hong Kong SAR China; ^3^ Department of Chemistry Hong Kong University of Science and Technology Hong Kong SAR China

**Keywords:** light emission, liquid crystal displays, photoalignment, quantum dots, quantum rods

## Abstract

Herein, emerging applications of luminescent semiconductor nanocrystals are addressed, such as quantum dots and quantum rods as down‐conversion materials used in liquid crystal displays (LCD). Their precisely tunable emission wavelengths and narrow emission bandwidths offer high color purity resulting in a wide color gamut with vivid colors for LCDs. Anisotropic materials, such as quantum rods, have the additional advantage of polarized emission, which can bring a significant improvement to the efficiency of LCD displays. The basic optical properties of these nanomaterials are considered, with a focus on quantum rods, and the challenges and progress in their assembly are discussed. Different techniques for quantum rod alignment are introduced such as shear‐oriented, electric field and magnetic field assisted assembly, mechanical rubbing, stretching, and electrospinning. The photoalignment approach allows for an easy arrangement of quantum rods in‐plane, and the implications of this method to patterning are considered. Different configurations of LCDs utilizing semiconductor quantum dots and quantum rods as down‐conversion layers are also presented, and the potential applications that are enabled by the wide range of emerging materials are highlighted.

## Graphical abstract

Herein, emerging applications of luminescent semiconductor nanocrystals are addressed with a focus on anisotropic quantum rods as down‐conversion materials for liquid crystal displays (LCDs). Techniques for the quantum rod alignment, with a focus on the photoalignment approach, and different configurations of LCDs are considered.

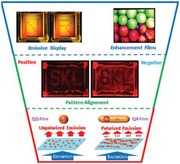

## 1 Introduction

Semiconductor nanomaterials, such as 0D quantum dots (QDs), 1D quantum rods (QRs), and 2D nanoplatelets offer high photoluminescence (PL) quantum yields (QY), saturated emission colors due to the reasonably narrow PL linewidth, and precisely tunable emission wavelengths based on both control of the composition and on the quantum confinement effect.^1^ These materials are thus considered nowadays as components for wide‐color‐gamut displays and light‐emitting devices (LEDs).^2^ There are two basic types of QD (or QR) LEDs: photoluminescent LEDs, relying on phosphors as color down‐converters such as a blue LED backlight unit (BLU) for liquid crystal displays (LCDs), and electroluminescent LEDs, where the active materials emit light as the recombination layer for injected charge carriers in a diode structure device.^3^ While there are still multiple issues which have to be resolved for the electroluminescent devices, such as balanced and efficient carrier injection and long‐term stability, photoluminescent LEDs have already been commercialized for the LCD backlight applications.^4^ Back in 2013, Sony released the first QD‐enabled display (Triluminous TV), in which the Color IQ optical subsystem, developed by the company QD Vision, was used to enhance the available color gamut of the LCD panels.[qv: 2b] QD Vision Inc. claimed that QD‐based LCDs are 25% more efficient in comparison to organic LEDs (OLEDs), and that LCDs equipped with QD enhanced backlights show a higher luminance.[qv: 4a,5] Later Samsung, TCL, and LG brought several flagship QD LCD TVs to their premium display markets.^6^ It has been estimated that the display market may grow to over USD $200 billion in the coming years, while the market for the required nanomaterials, primarily QDs, was estimated to grow to over USD $10 billion by 2020.^7^ Touch Display Research Inc. predicted that by 2025 more than 60% of TVs and over 50% of monitors may adopt semiconductor QDs and related materials.[qv: 4a] Additionally, they estimated that the total market for QDs was worth more than USD $7.0 billion in 2017 and is expected to grow up to USD $10 billion by 2020.[qv: 4a] This is comparable to the OLED market size.^6^, ^8^ The use of QDs in displays is thus maturing, and there are many opportunities for new concepts and innovative device designs, while the search for better light‐emitting materials is on‐going. Initial commercial products (such as the Sony TV) used CdSe‐based QDs, as the most well studied kind of QDs, and also because these QDs exhibit solid performance in terms of color quality and high PL QY. However, environmental concerns and governmental regulatory restrictions limiting the use of the toxic element cadmium, have hindered sales of those products in some markets. InP‐based QDs have been explored in the Samsung QD TVs, due to the reduced toxicity and thus the unrestricted use of this material in consumer products in most markets. However, indium is a rare element which is already heavily used in other applications, and the color saturation of InP QDs does not match that of CdSe QDs, due to their wider emission linewidth. Lead halide perovskite nanocrystals^9^ have recently emerged as another promising light‐emitting material for displays, due to their ease of synthesis and exceptionally narrow emission linewidths. Unfortunately, the large degree of ionic character in these materials limits their stability, this in addition to the presence of toxic Pb, may become a limiting factor for commercialization. For the older class of II‐VI materials, both 1D QRs and 2D nanoplatelets offer an added advantage for LCDs, because they can emit linearly polarized light.^10^ At the same time, those anisotropic non‐spherical nanocrystals require a proper processing, as they need to be assembled with a high degree of orientational order so as to gain the benefit of the linear polarized emission as an ensemble.

This review offers a comprehensive discussion of the potential use of down‐conversion colloidal nanomaterials for LCD applications. After introducing basic optical properties of QDs and QRs, we will consider various assembly strategies for QRs, with a particular emphasis on assembling anisotropic materials to obtain polarized emission in the ensemble. Finally, we will present QD‐ and QR‐based device architectures and highlight several concepts that emerge from the wide variety of light‐emitting materials that are now feasible for these applications.

## 2 Basic Optical Properties of Semiconductor Nanocrystals

### 2.1 Quantum Dots

**Figure 1:**
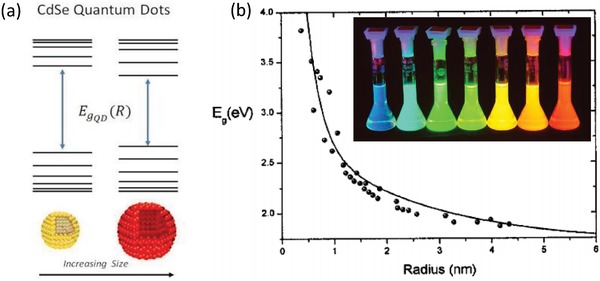
a) Schematic illustration of the band structure of CdSe QDs of two different sizes. b) Energy bandgap dependence on the radius of CdSe QDs, here dots are the experimental data and the solid line is the calculated data using Equation (1). The inset shows photograph of core/shell CdSe/ZnS QDs of different sizes (synthesized at University of Hamburg in 2001) emitting all over the visible spectral range. Reproduced with permission.^11^ Copyright 2006, American Institute of Physics.

The term “quantum dot” has been given to spherical (or quasi‐spherical in case of wurtzite materials such as CdSe) semiconductor nanocrystals because they exhibit quantum confinement effect, for nanoparticles with radii smaller than the Bohr radius of the bulk exciton.^1^ At that point, the bandgap of the QDs becomes size‐dependent, increasing with a decreasing radius due to the increased interaction energy between the hole and the electron. As an example, when the radius of CdSe QDs decreases from 4.15 to 0.60 nm, the photon energy of the first absorbance peak, *E*_12_, increases from 1.88 to 3.02 eV (the bandgap energy of bulk CdSe is *E*_g_ = 1.7 eV), and the emission color changes from red to blue.^11^ The most basic relationship between the band gap of a QD *E*_gQD_ and its radius, *R* is based on the effective mass approximation and includes the Coulomb interaction, as given by the so‐called “Brus equation”:^12^

(1) $$E_{\mathrm{gQD}}\left(R\right)=E_{g}+\frac{1}{R^{2}}\left(\frac{m_{o}}{m_{e}}+\frac{m_{o}}{m_{h}}\right)\frac{h^{2}\alpha_{n,l}^{2}}{8\pi^{2}m_{o}}-\frac{1}{R}\left(\frac{1.8e^{2}}{4\pi \varepsilon_{o}\varepsilon_{r}}\right)$$

where *E*_g_ is the bulk band gap energy, *h* is Plank's constant, *m_o_* is the rest mass of an electron, *m*_e_ and *m*_h_ are the effective masses of electrons in the conduction band and holes in the valence band, respectively, and α_*n*,*l*_ is a dimensionless series that takes discrete values. A simple illustration of the size dependence of the electronic structure of prototypical CdSe QDs, together with their band gap dependence on average radius of QDs resulting in different colors of emitting light is given in **Figure** **1**.

Cadmium chalcogenide nanocrystals have been by far the most common QD materials for the emission in the visible spectral range, spanning it from the blue (CdS) to cyan, green, yellow, orange, and red (CdSe), and extending further into the deep red for CdTe.^1^ For the cadmium free alternatives, there are ZnSe QDs for emission in the blue, and InP and CuInS_2_ for the visible spectral range.^1^ For II‐VI QDs, high PL QYs often become accessible with the growth of an epitaxial inorganic shell, leading to the formation of so‐called Type I core/shell particles. Depending on the band gap alignment of these materials, such heterostructures are commonly denoted as Type I or Type II.^13^ Type I band alignment describes a core/shell system where the band gap of the core is smaller than the band gap of the shell material, and both the conduction and the valence band edges are higher/lower for the shell material as compared for the core material. A typical example for the Type I core/shell QDs are CdSe/ZnS nanocrystals, where the shell keeps excitons in the core and at the same time efficiently passivates surface traps, which results in enhanced stability and high PL QYs (see inset in Figure 1b). While PL QY is one of the main factors for the QDs used for down‐conversion, the spectral linewidths of the emission peak, measured as the full width at half maximum (FWHM), is equally important for display applications. Narrow emission peaks indicate good size homogeneity in an ensemble of QDs and ensure high color purity of the emitters, translating into a wide range of accessible, vivid colors for the devices employing red‐green‐blue (RGB) mixed colors.

### 2.2 Quantum Rods

Semiconductor QRs^14^ are 1D nanomaterials which, unlike 0D QDs, offer the added advantage of polarized emission. Polarized emission sources are able to significantly improve the optical efficiency of LCDs since only photons with their electric field aligned parallel to the LC polarization can pass through the device. A related material set is the 2D nanoplatelets,^14^, ^15^ which are not considered here, but have been reported with polarized edge emission, though these are more difficult to align in an LCD.^16^ Anisotropic optical properties in QRs, including an increased absorption along the long axis of the rod, and partially polarized emission can be explained by the anisotropic electric field strength along different dimensions. It has been established that a structure with an anisotropic shape will attenuate electric fields perpendicular to the long axis.[qv: 15b] However, polarization in CdSe/CdS dot‐in‐rods and rod‐in‐rods goes beyond what can be explained by simple dielectric effects. The additional anisotropy originates from the splitting of the exciton fine structure in the wurtzite CdSe core.^17^

**Figure 2:**
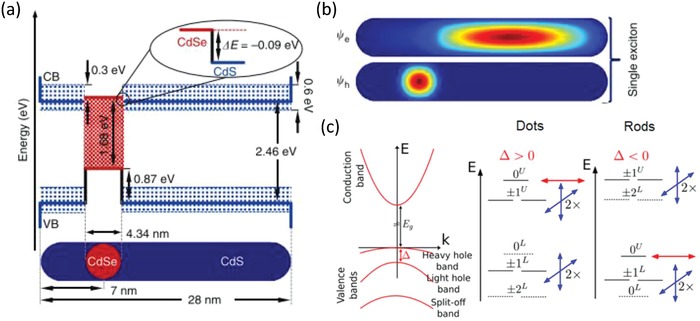
a) Schematics of a CdSe/CdS dot‐in‐rod nanostructure, along with the interfacial band alignment of the CdSe and CdS constituent materials. The core diameter (4.34 nm) and the CB offset (0.09 eV, inset) correspond to the values adopted for the calculation of the electronic wave functions and lasing wavelengths. The values of the VB offset (0.87 eV) along with those of the bandgaps for CdSe (1.68 eV) and CdS (2.46 eV) are also shown. The blue shadowed area indicates the range of potential values for the CB offset. Calculated wave functions for the electrons and holes for the laser emission in b) the single‐exciton regime at 628.6 nm, exhibiting mixed carrier dimensionality and small overlap, indicating that for the first exciton the hole/electron wave functions are localized in/near the core. Reproduced with permission.[qv: 10b] Copyright 2013, Nature Publishing Group. c) Band structure of CdSe (left), and band‐edge exciton fine structure energy states for a positive (∆ > 0) or negative (∆ < 0) net‐splitting (right). Reproduced from permission.[qv: 17b] Copyright 2015, American Chemical Society.

It was reported that the emission from a semiconductor nanoparticle becomes partially polarized when the aspect ratio of the core exceeds 1.2:1.^10^, ^18^ The polarized emission from a QR was explained on the basis of the fine structure splitting, shared 2D excitation as shown in **Figure** **2**a,b, the level ordering and the oscillator strengths of the various transitions, which depend on the elongated shape. A detailed related discussion is given in ref. [qv: 17b]. The spherical symmetry of the dot‐like core should lead to a band‐edge exciton structure with the same symmetry as for the spherical nanocrystals, which show a low degree of polarization (DOP) for the emission. This is in contrast with the observed strongly polarized emission, which indicates that the anisotropic shell around the core can modify the electronic structure, as was reported, for example, in ref. [qv: 17b] for CdSe/CdS dot‐in‐plate structures. In the excited state, the electron wavefunction from the CdSe core is able to extend into the conduction band of the CdS shell, and into the shell volume to create polarized emission.[qv: 10b]

**Figure 3:**
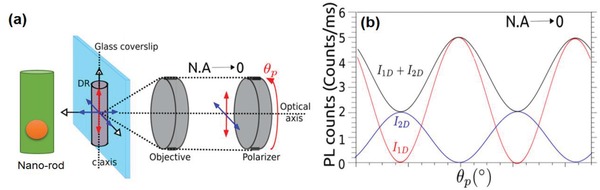
a) Illustration for the three oscillators and b) the emission from 1D (red line) and 2D excitons (blue line) of a core/shell CdSe/CdS dot‐in‐rod structure, that results in the emission of light (black line) with polarization azimuth parallel to the c‐axis of the QR. Reproduced with permission.[qv: 17b] Copyright 2015, American Chemical Society.

Vezzoli et al. compared energy level diagrams for both quantum dots and rods, as shown in Figure 2c.[qv: 17b] The left part of Figure 2c presents the band structure of CdSe, showing the heavy‐hole, light‐hole, and split‐off sub‐bands and the net‐splitting Δ at *k* = 0. The middle part of Figure 2c demonstrates the band‐edge exciton fine structure energy states for a positive net‐splitting Δ. The | ± 2〉 states and the |0^*L*^〉 states are optically inactive and represented as dashed lines. The degenerate 2D dipole emission from the | ± 1^*L*^〉 and | ± 1^*U*^〉 levels is symbolized by the double blue arrows. The 1D dipole emission from the |0^*U*^〉 state is symbolized by the red arrow. The right part of Figure 2c illustrates the band‐edge exciton fine structure energy states for a negative net‐splitting Δ, showing a swapping of the levels. The polarized emission in these figures can be illustrated as follows. The valence band of CdSe is made of 3 sub‐bands, the heavy‐hole, light‐hole, and split‐off sub‐bands (Figure 2c). The | ± 2〉 and |0^*L*^〉 state do not contribute to the emission at the room temperature, when it is a mixture of recombination from the |0^*U*^〉 state and from the degenerate | ± 1^*L*^〉, | ± 1^*U*^〉 states.^18^ The |0^*U*^〉 state is associated with a linear 1D dipole that oscillates along the *c*‐axis of the crystal and emits linearly polarized photons. The | ± 1^*L*^〉 and | ± 1^*U*^〉 can be seen as 2D dipoles, oscillating inside a plane, as schematically shown in **Figure** **3**a. Because of the level degeneracy, the emission from these transitions is an incoherent superposition of σ^+^ and σ^−^ components,^19^ and the corresponding dipole is called a degenerate 2D dipole. It is equivalent to two linear dipoles, oscillating perpendicularly and in quadrature. These dipoles are contained in the plane perpendicular to the *c*‐axis of the crystal and because of this the polarization azimuth for the two oscillators are mutually perpendicular as illustrated in Figure 3b. Therefore, the degree of the polarization for the total emission strongly depends on the relative oscillator strengths of two oscillators mutually oscillating in an orthogonal plane.

**Figure 4:**
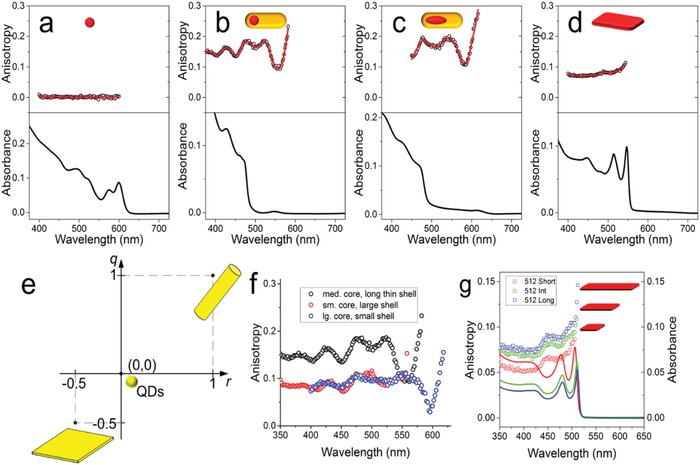
Anisotropy (open circles, red curve) and absorption (black curve) of several colloidal nanostructures: a) CdSe sphere (5 nm); b) CdSe/CdS dot‐in‐rod (5.1 × 35 nm; 3.8 nm core); c) CdSe/CdS rod‐in‐rod (5.2 × 45 nm; 3.8 × 9.0 nm core); d) CdSe nanoplatelet. e) A scheme showing absorption and emission polarizations attainable for ideal dots, rods and platelets. f) Comparison of optical anisotropy in different samples of CdSe/CdS dot‐in‐rods with varying core and shell dimensions. Medium core, long thin shell corresponds to 5.1 × 35 nm shell with a 3.8 nm core. Small core, large shell corresponds to 6.5 × 12 nm shell with a 2.3 nm core. Large core, small shell corresponds to 4.9 × 18 nm with a 4.6 nm core. g) Solution anisotropy (open squares) and absorbance (solid lines) of three samples of CdSe nanoplatelets with identical thickness and different lateral dimensions: 3.9 × 8.8 nm for “short,” 7.1 × 17 nm for “medium” and 8.7 × 29 nm for “long.” Reproduced with permission.^14^ Copyright 2015, American Chemical Society.

The core size, the shell thickness, and the aspect ratio affect the polarized emission properties of core/shell QRs.[qv: 14,17b,20] For larger core sizes, wurtzite CdSe cores tend to grow into a prolate shape, which strongly enhances optical anisotropies. Due to the shape dependence of the energy level structure, the degree of the polarization for the emitted light increases with the aspect ratio of the QRs, and it is further increased by the anisotropic dielectric environment.^21^ Murray's group has shown that the thickness of the shell along the core is critically important to achieve a higher degree of polarization.[qv: 20b] Although a rod‐shaped shell geometry is a necessary condition to observe the polarized optical properties, the degree of polarization is not a direct consequence of the increasing aspect ratio. Rather, tuning the local anisotropy of the emissive core materials by changing the thickness of the anisotropic shell may change the degree of optical anisotropy more dramatically.[qv: 20b] Thus, the degree of polarization depends on first, on the fluorescence anisotropy of the nanoparticle and second the quality of alignment. Talapin's group studied the anisotropy for different shapes of colloidal semiconductor nanostructures, including dots, rods, and platelets, all illustrated in **Figure** **4**.^14^ Among these different types of nanostructures, QRs show enhanced absorption and emission of light along their long axis.[qv: 17b,20b,22] Furthermore, among the core/shell elongated nanoparticles, dot‐in‐dot and rod‐in‐rod heterostructures have higher anisotropy in comparison to the other nanoparticles. Particularly, the rod‐in‐rod nanoparticles show the highest anisotropy. Thus, these heterostructures show a high degree of linear polarization for emission and are highly suitable for modern LCDs. Figure 4e shows the size‐dependent anisotropy for the nanoparticles. The wavelength‐dependent anisotropy for various core/shell structures has been plotted in Figure 4f. It is clear from the figure that the anisotropy increases close to the band edge. However, it is not very suitable for LCD backlight applications, where a fixed blue light is used to excite the PL layer. The same figure also conveys that the thin shell and medium size core provide a higher anisotropy.

### 2.3 Alignment Techniques for Quantum Rods

The alignment of QRs with a high order parameter is critically important, and several techniques have been explored in this regard, including evaporation‐mediated assembly,^23^ electric field‐assisted assembly,^24^ template‐assisted assembly,^25^ LC self‐alignment,^26^ Langmuir–Blodgett deposition,^27^ and mechanical rubbing.^28^ Several other, less significant methods have also been investigated.^29^ While most of these methods control the assembly of QRs by an external force, for the most part they offer limited flexibility for the local alignment orientation, which reduces their prospects for the type of large‐scale fabrication required for LCD. In contrast, electrospinning^30^ and stretching of polymer films^14^, ^31^ have recently achieved significant advantages in this respect. Some of the approaches allowing for large area alignment of QRs are discussed below.

#### 2.3.1 Shear‐Oriented Assembly

**Figure 5:**
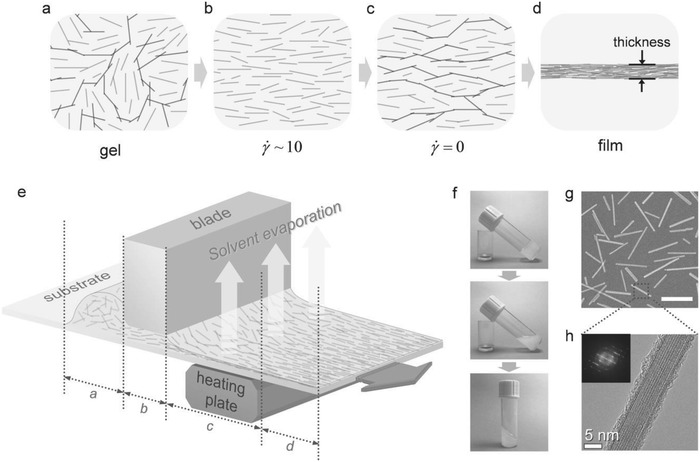
Snapshots of QRs in the thixotropic suspension at each step of the coating process: a) gel with random networks, b) sheared sol where the networks break down, c) gel with aligned networks after shear, and d) aligned film after solvent evaporation. e) Schematic illustration of the fabrication of aligned QR‐based thin films by the blade‐coating process. f) Thixotropic behavior of the LaPO_4_ QR gel suspension: top, gel; middle, sol after stirring; bottom, gel after setting the sol inclined. g) SEM image of the LaPO_4_ QRs (the scale bar indicates 200 nm). h) High resolution transmission electron microscopy image and its fast‐Fourier‐transform (FFT, inset) of a single crystalline LaPO_4_ QR. Reproduced with permission.^31^ Copyright 2013, Wiley‐VCH.

Gacoin's group has developed a simple coating method to make an aligned QR film using a thixotropic gel suspension, as illustrated in **Figure** **5**.^31^ A schematic of their customized coating machine is shown in Figure 5e. A 20 µL drop of solution containing QRs was deposited onto a substrate and the substrate was dragged under the coating blade by a robotic arm with a constant speed and a fixed gap thickness from the blade plane. The gap thickness was varied from 20 to 100 µm to control the film thickness. The substrate, coated with the sheared suspension coming out of the blade, was heated to 140 °C on a hotplate to evaporate the ethylene glycols solvent. Later the solidified film was annealed in a furnace at 500 °C for 2 h. While dragging the blade over the suspension, a constant shear rate (d*y*/d*t* ≈10 s^–1^) was applied after generating a Couette flow in the gap and thus uniformly shearing the suspension. They confirmed that the required rate for the QR orientation in the rod gel is only ≈10 s^–1^, which is suitable for the typical coating techniques used for roll‐to‐roll processes. When the flow stopped, the attractive interparticle forces led the sol to turn back to a gel state in the absence of shear stress. This froze the rods into an oriented alignment, with only minor short‐range defects present. This approach was used to align QRs having relatively large size (length ≈200 nm) compared to CdSe‐based QRs normally used. However, high temperature processing makes it difficult to be used for LCD applications.

#### 2.3.2 Electric Field Assisted Assembly

**Figure 6:**
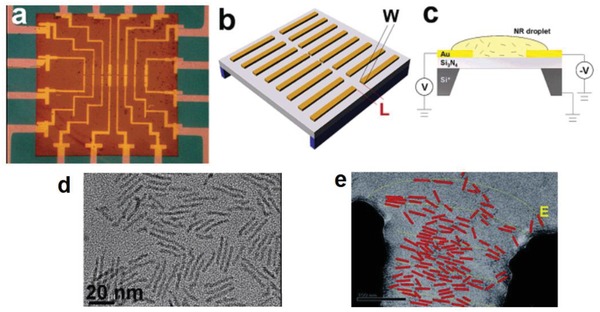
a) Optical micrograph of a typical device used for the electric field induced alignment, containing eight electrode pairs on a 80 × 80 µm^2^ Si_3_N_4_ membrane window (copper‐colored square). b) Schematic of a device showing the suspended membrane containing eight patterned electrode pairs with different gap widths *W* (0.5–20 µm) and lengths *L* (0.1–1.1 µm). c) Schematic of the alignment experiment: voltage is applied to the electrodes, while a drop of solution containing QRs dries on the surface. The p‐doped Si layer is grounded. d) TEM image of randomly oriented 38 nm × 3.4 nm CdSe QRs, used in that study, imaged on a holy carbon grid. e) TEM image of aligned QRs, artificially colored in red. Reproduced with permission.^24^ Copyright 2006, American Chemical Society.

Drndic's group reported the alignment of CdSe and CdTe QRs using applied electric fields.^24^ The devices used for the alignment consisted of an interdigitated electrode on an approximately 100 nm thick silicon nitride (Si_3_N_4_) to hold the suspended membranes, as shown in **Figure** **6**. CdSe or CdTe QRs (10 µL) in a hexane/octane solution were drop‐casted onto each device while voltage was applied to the electrodes. Local electric fields generated by nanopatterned electrodes were used to control the position and orientation of both well‐isolated and closely packed QRs. Post‐deposition imaging using TEM and atomic force microscopy revealed QR alignment in the direction of the applied field, and dense accumulation around and directly on the voltage‐biased electrodes when deposited from dilute and concentrated solutions, respectively. The degree of alignment under the applied electric field was characterized by a nematic order parameter *S* ≈0.8 in contrast to the zero‐field case where *S* was ≈0.1. Overall, this approach appears to work well, but it is not feasible for the large‐scale alignment of QRs, required for LCD application, due to the necessity of having pre‐printed electrodes on the substrate during deposition.

#### 2.3.3 Magnetic Field Assisted Assembly

Pietra et al. studied spontaneously self‐assembled aggregates in a suspension of the CdSe/CdS QRs.^32^ The related measurements addressed the magnetizability α^*M*^ of a single QR, defined by *m* = α^*M*^ *B*, where *m* is the magnetic dipole moment that is induced by a magnetic field *B*. Because of demagnetization effects, the magnetizability of a QR was more negative in the transverse direction $\alpha_{⊥}^{M}$ than in the longitudinal direction $\alpha_{∥}^{M}$. The overall response of ordered and oriented QRs to external fields could be calculated from the magnetic susceptibility of the material and the environment.^32^

#### 2.3.4 Self‐Assembly at the Liquid/Air Interface

**Figure 7:**
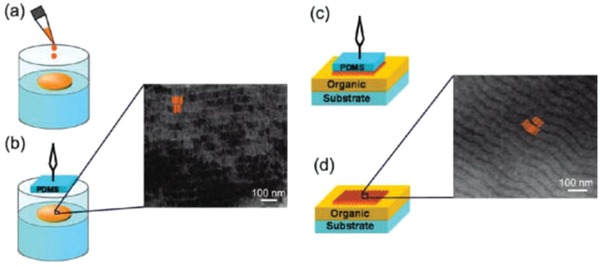
A sketch of the technique developed for the deposition of ordered QR arrays on organic layers. a) A toluene solution of QR is first spread on water. b) After the solvent evaporation, the floating film is finished by means of a PDMS stamp pad or a carbon coated TEM grid; the zoom displays a TEM image of a self‐assembled QR film on the latter substrate. QRs are arranged in ribbon‐like structures in which they are joined side‐by‐side along their elongated *c*‐axis, thereby resembling the smectic phase of a liquid crystal. c) The inked PDMS is brought into conformal contact with the organic layer by applying a gentle pressure. d) Finally, the stamp is removed, and the QR film is successfully transferred onto the organic surface. The zoom shows a scanning electron microscopy (SEM) image of such laterally aligned QR film. Some of QRs are highlighted in orange as a guide to the eye. Reproduced with permission.[qv: 33a] Copyright 2009, American Chemical Society.

By accurately controlling the concentration of QRs in the solution and by slowing down the solvent evaporation process, it is possible to prepare films of close‐packed QRs floating on the surface of water organized in nematic and/or smectic phases, as shown in **Figure** **7**. The tendency toward self‐organization of monodisperse QRs can be understood in terms of surface tension balance. When a small volume of a concentrated organic solution of QRs is spread onto a water surface, slow and uniform solvent evaporation leads to a continuous increase of their concentration in the remaining droplet. As soon as the solvent evaporates, hydrophobic and dipolar interactions among the surfactant coated QRs are progressively enhanced due to their gradually increasing concentration in the organic phase and thus shortening of the interparticle distance. Such intermolecular forces induce segregation of the QR in close‐packed lateral ribbon superstructures or rod/raft aggregates, as a means of decreasing the overall surface energy and interfacial energy of the system.^26^, ^27^, ^33^ However, because of poor control over the in‐plane orientation of the QRs and the limited size of the film, it is very difficult to apply this method for LCDs.

#### 2.3.5 Mechanical Rubbing

**Figure 8:**
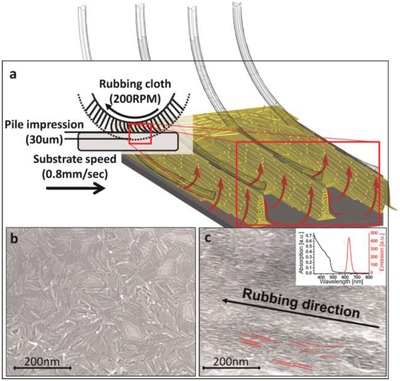
a) Schematics of the rubbing process—a rubbing cloth is pressed against a spin‐coated thin film and the rotation of the fibers oppositely to the movement of the sample forms trenches and lines as the fibers push the QRs piling them up in oriented stacks. b) SEM images of a spin‐coated thin film of CdSe/CdS QRs shows no preferred directionality. c) SEM of the rubbed QR shows an alignment parallel to the rubbing direction. The inset shows absorption (black) and emission (red) spectra of the QRs. Reproduced with permission.^28^ Copyright 2012, Wiley‐VCH.

Amit et al. reported the use of mechanical rubbing for the macroscopic scale alignment semiconductor QRs.^28^ The CdSe/CdS QRs, exhibiting linearly polarized emission, were aligned by mechanical rubbing of a spin‐coated glass substrate, as shown in **Figure** **8**. The dragging force exerted by the rubbing fibers resulted in deflection and reorientation of the QRs along the rubbing direction. The rubbing process was optimized by tuning surface–particle and particle–particle interactions yielding high polarization ratios of 3.5:1 relative to the previously reported values of 1.75:1 and 2.2:1 attained by the rubbing of the QR layers. The surface–particle interaction is an important factor for the rubbing process, one that may have serious limitations on the thickness of the film. The film thickness (at least several microns, depending on the QR concentration and absorption coefficients) should be enough to maintain color saturation and brightness for LCD applications.

#### 2.3.6 Stretching of Polymer Films

**Figure 9:**
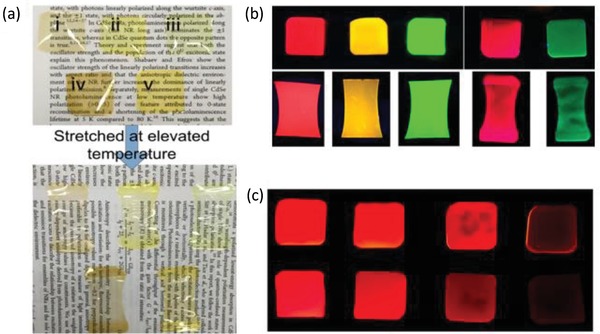
a) Films consisting of red‐luminescent CdSe/CdS QRs (i, ii, iii) and nanoplatelets (iv, v) dispersed in poly(butyl‐*co*‐isobutyl methacrylate) under ambient light before (top) and after stretching (bottom). b) Same films under UV light before (top) and after stretching (bottom). c) Films with varying concentrations of QDs (top row) and QRs (bottom row) in the polymer films under UV light. Reproduced with permission.^14^ Copyright 2015, American Chemical Society.

The stretched film approach has been reported in detail by Cunningham et al, and is illustrated in **Figure** **9**.^14^ It is very simple and offers good uniformity of the resulting samples, but significant stretching is required. CdSe/CdS dot‐in‐rods were dispersed in polymer films of polybutyl‐*co*‐isobutyl methacrylate. In non‐stretched films, QRs were randomly oriented, while a strong unidirectional alignment was observed after stretching of the composite films. A high degree of alignment was achieved, corresponding to an orientation factor of 0.87, and large areas of the films demonstrated polarized emission, with the polarization ratio (*I*_max_/*I*_min_) of ≈5.6:1.

#### 2.3.7 Electrospinning

**Figure 10:**
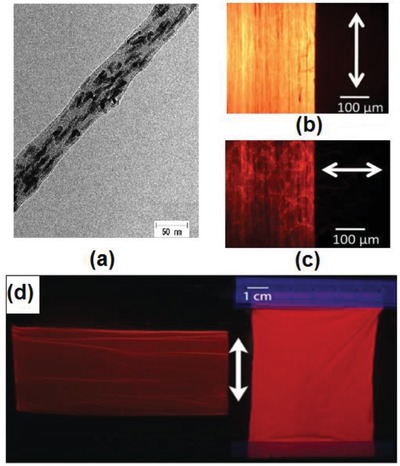
CdS QRs aligned in the nanofiber sheets by electrospinning. a) TEM image of a nanofiber with aligned QRs. The diameter of QRs is ≈5 nm and length is ≈40 nm. Frames (b) and (c) represent polarization microphotographs of a nanofiber sheet (b) when the polarizer is parallel to the emission polarization and c) when the polarizer is perpendicular to the emission polarization. d) The aligned QR nanofiber sheets. The white arrow indicates the polarization direction, which is perpendicular to the longitudinal direction of the sheet shown on the left‐hand side, and parallel for that on the right‐hand side. Reproduced with permission.[qv: 30a] Copyright 2015, American Institute of Physics.

The electrospinning uses a high electric field to fabricate nanofiber bundles, which are collected by a high‐speed rotating disk and drum, producing large sheets. The diameter of the electrospun nanofibers mainly depends on the polymer concentration in the dispersed QR solution used for the electrospinning process. The rotation speed of the drum controls the alignment of the nanofibers in the sheet. The electrospinning approach was first proposed for the alignment of the gold nanorods,^34^ and later was applied to light‐emitting semiconductor CdS QRs and quantum wires.^30^ Recently, the company Merck has used the same approach to make nanofiber bundles and sheets containing QRs.[qv: 30a] Various aspects of nanofiber processing have been studied, such as the polymer concentration dependence of the diameter of the electrospun nanofibers and effect of the rotation speed of the drum on the alignment quality of QRs. Merck has achieved good polarization ratio for the 6 × 10 cm^2^ QREF, which is characterized by high uniformity and DOP of 0.6, see **Figure** **10**. This approach appears to be useful to produce highly uniform QR films; though, strong electric fields and somewhat complicated fabrication process impose hurdles on the commercialization of this method.

#### 2.3.8 Photoalignment

Photoalignment technology offers precise control at the nanoscale for the planar alignment of LCs and LC polymers.^35^ The anchoring energy is a force applied from the alignment layer that holds the LC molecule in the desired position, in the absence of external stimuli. Photoalignment offers a large anchoring energy (≈10^–3^ J m^−2^) with a high in‐plane, easy axis control providing a small pre‐tilt angle (<0.2°). This method also offers high spatial alignment resolution,[qv: 35c,36] and facilitates QR alignment in LC polymers.[qv: 35b] Most often, a sulfonic azo‐dye tetrasodium‐5,5′‐((1E,1′E)‐(2,2′‐disulfonato‐[1,1′‐biphenyl]‐4,4′‐diyl)bis(diazene‐2,1‐diyl))bis(2‐hydroxy‐benzoate) (SD1 dye) has been used as the photoalignment layer.^37^ When the SD1 layer is irradiated by polarized light with a wavelength of 450 nm, the azo‐dye molecules that have their transition dipole moments parallel to the electric field (E‐) vector of the impinging light receive excess energy. This results in their reorientation from the initial position to a direction orthogonal to the E‐vector of the irradiating light which defines the alignment direction (i.e., the easy axis) perpendicular to the E‐vector of the irradiating light, with almost zero tilt angle and a high anchoring energy.^37^

**Figure 11:**
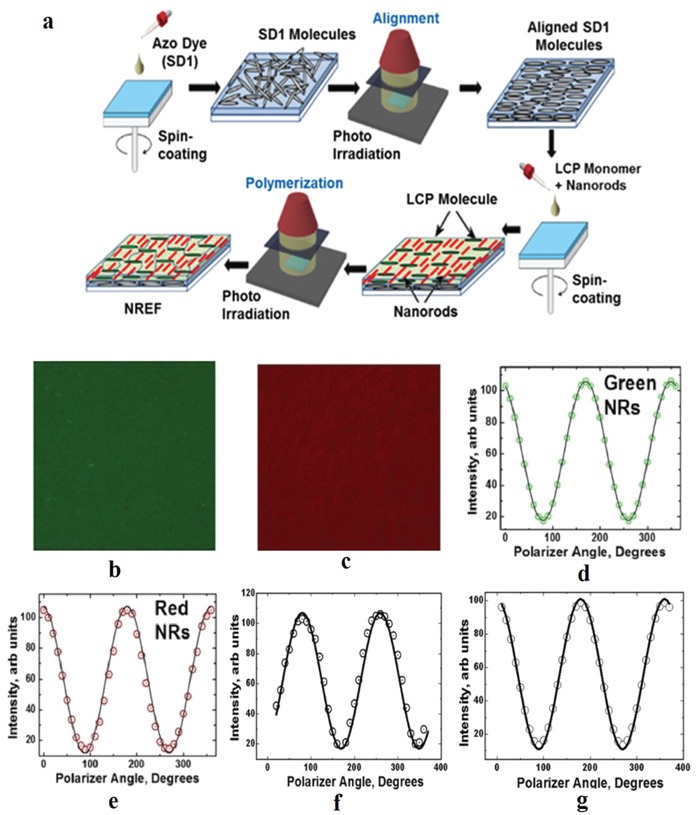
a) Process flow to fabricate QREFs by photoalignment technology. b) 250 × 250 µm^2^ fluorescence micrographs of green (b) and red (c) emitting QREFs. The corresponding curves for the emitted intensity as a function of the polarizer axis rotation angle, are presented in section (d) for green emitting QR and (e) for red emitting QR. Frame (f) represents the mixed QREF and (g) the multiple layer QREF. Reproduced with permission.^39^ Copyright 2017, Wiley‐VCH.

The fabrication of photoaligned LCP/QR films is a multi‐step process which represents a straightforward and easily scalable procedure, as shown in **Figure** **11**a. The SD1 alignment layer transfers a torque to the LC molecules and aligns them in a direction parallel to the easy axis of the SD1.[qv: 35a,38] Simultaneously, due to the repulsive intermolecular forces between the QR ligands and LC molecules, which exert counter‐torque on QRs, QRs are aligned perpendicular to the easy axis of the SD1. The QR ligands, to minimize the total energy of the system, try to fit in the monomer matrix like a comb. This provides a long‐range order to the QRs.[qv: 35b] The order parameter of the resulting QR‐based composite films calculated from the TEM images could be as high as 0.87.[qv: 35b] Examples of such QR enhancement films (QREFs) are shown in Figure 11b and c for the green and red emitting QRs, respectively, confirming the microscopically uniform distribution of QRs without any manifestation of clustering.

The polarization angle dependence of the emitted light for green and red emitting QREFs are presented in Figure 11d and e, respectively, and show good agreement with the Malus' law *I* (θ) = (*I*_max_ − *I*_min_) cos^2^θ + *I*_min_.^40^ The black line representing Malus' law fits well with the experimental data represented by the open circles for both rods. The measured polarization ratio for the aligned red and green QR were 7.8:1 and 7:1 respectively, confirming the high‐quality alignment of QRs in the QREF.[qv: 17b,20b,41]

QRs in an QREF for the LCD backlight application have to be arranged in a suitable configuration to provide the desired white balance of the backlight unit. There are two possible alternatives: first, mixing both types of QRs in the same film and optimizing the concentration. In this configuration, it is particularly important for the green emitting QRs to compensate for the reabsorption of the green emission by the low energy emitting QRs. Second, making a multiple layer stack and arranging the high‐energy emitters on top of the low energy emitters. Both of these alternatives were studied by Srivastava et al.^39^ The DOP of the mixed QREF and multiple layer QREF were measured as 0.67 and 0.76, obtained from Figure 11f and g, respectively. Based on these results, one can conclude that the multiple layer configuration is better. However, for LCD backlight applications, there are two scaling factors for the QREF brightness, first, the PL QY for the QRs, and second, the DOP of the aligned QREF. Thus, to enhance the LCD performance, both the PL QY and the DOP should be optimized.

#### 2.3.9 Patterning by Photoalignment

**Figure 12:**
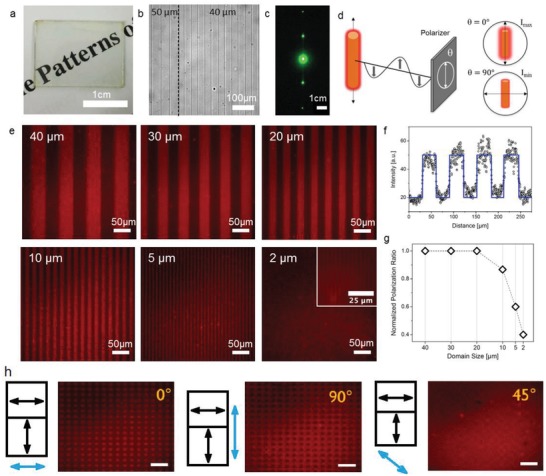
Pattern photoalignment of the light‐emitting CdSe/CdS QRs. a) Substrate containing six grating patterns with differing pitch/domain sizes in ambient light. b) Optical microscope images of the same substrate, presenting two neighboring grating patterns separated by a dashed line with domain sizes of 50 (left) and 40 µm (right). c) Diffraction image of a green laser (543.5 nm) after passing through the diffraction grating, showing zero, first and second order diffraction. d) Schematics, highlighting the origin of dark (*I*_min_) and bright (*I*_max_) states, according to the orientation of the QRs in regard to the polarizer main axis. e) Micrographs of fluorescent grating patterns with domain sizes of 40, 30, 20, 10, 5, and 2 µm. f) Representative intensity profile, recorded from the 30 µm pattern, following Malus' law. g) Normalized polarization ratios (from 2.5 to 1), determined from the intensity profiles (panel (f)) of each grating pattern, shown in (e). Format is chosen to illustrate the relative drops in the polarization ratios. The films were excited with a 12 V/100 W halogen lamp through a 546/12 nm bandpass filter. h) A simple illustration of the 2D binary alignment of the QR and emission profile against the polarization azimuth of the irradiating light. Reproduced from permission.[qv: 35c] Copyright 2017, American Chemical Society.

In addition to uniform alignment, the photoalignment approach offers the possibility to align the light emitting QRs on patterned surfaces.[qv: 35c] The azo dye‐based alignment layer has the ability to reorient the alignment direction upon subsequent irradiation by polarized light with a different polarization azimuth. This allows us to realize multi‐domains alignment of QRs: by adding an amplitude mask for the second irradiation, a micro‐scale alignment pattern for the QRs can be achieved with a distinguishable easy axis of alignment. Using this technique, domain sizes as small as 2 µm could be realized for the binary alignment domains, as shown in **Figure** **12**.

To summarize the section on different alignment techniques, all of the above approaches, except for the photoalignment method, provide QR arrays with poor in‐plane control, limiting the highest reported DOP to 0.64 for CdSe/CdS dot‐in‐rod nanostructures.^14^, ^23^, ^24^, ^25^, ^26^, ^27^, ^28^, ^29^, ^30^, ^42^ Theoretically, the maximum DOP that could be achieved for such QRs, which are uniformly aligned in‐plane with a high order parameter, is around 10:1. For LCD BLU applications, there are four approaches that are most suitable for device manufacturing, namely photoalignment, electrospinning, polymer stretching of composite films, and the electric field assisted assembly, whose major characteristics are compared in **Table** **1**. The photoalignment, stretching and electric field assisted approaches show good order parameter, but only photoalignment provides perfect in‐plane alignment control. Electrospinning gives superior uniformity but the order parameter is limited to 0.6 only. Photoalignment shows high DOP, high order parameter and acceptable film uniformity.

**Table 1:** Comparison of the reported characteristics of QR‐based oriented films produced by four different alignment techniques potentially suitable for the LCD BLU fabrication

	Photoalignment	Electrospinning	Polymer stretching	Electric field assisted
Degree of polarization	≥0.76	≈0.6	0.7	0.6
Order parameter	≥0.85	N/A	0.8	0.8
In‐plane orientation	Yes	No	No	Depends on the electric field
Polarization ratio	≥7.8:1	4:1	6:1	4:1
Brightness	≥300 nits	N/A	N/A	N/A
Uniformity	Acceptable	High	Acceptable	Poor
Pattern alignment	Possible for multiple domains	Not possible	Not possible	Possible
Compatibility with LCDs	+++	++	++	+

## 3 Use of Quantum Dots and Aligned Quantum Rods in LCDs

### 3.1 Advantages and Limitations of the Contemporary LCDs

**Figure 13:**
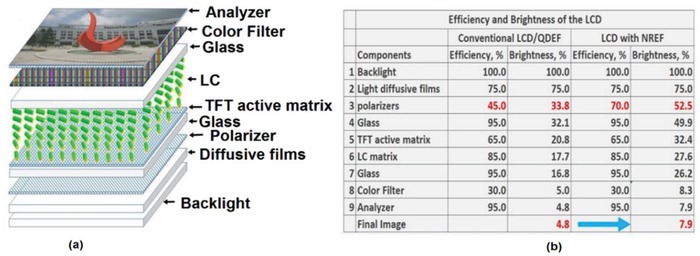
a) The common LCD architecture, with an indication of the constituent elements. b) A table listing LCD components with their optical efficiencies and brightness. Reproduced with permission.^39^ Copyright 2017, Wiley‐VCH.

The burgeoning field of flat panel displays now encompasses both inorganic and organic electroluminescent LEDs;^43^ nevertheless, LCDs have maintained their dominant position in this field. The main advantages of LCDs are their low power consumption, low driving voltages, and cheap fabrication. There has been a vivid discussion in the display technology field on the topic “*LCD versus OLED (organic light emitting diodes), who wins?*”^44^ Both technologies have their own merits. Generally speaking, LCDs are leading in lifetime, brightness, and manufacturing cost, and they are comparable to OLEDs in resolution density, power consumption, ambient contrast ratio, and viewing angle but inferior to OLEDs in black state, panel flexibility, color gamut, and response time. However, recently introduced QD backlight technology has narrowed the performance gap between the LCD and OLED^44^, ^45^ and offered wider color gamut and lower power consumption. Furthermore, local dimming boosts the dynamic contrast ratio to 1 000 000:1.^46^ The remaining challenge for LCDs is the response time, which is ≈100 times slower than in OLED. In reality, this is only a superficial challenge due to the limitation imposed by the addressing time of the thin film transistors. A range of approaches to improve the response time versus polymer‐stabilized blue phase LCs,^47^ low viscosity nematic LCs,^48^ and ferroelectric LCs^49^, ^51^ have been suggested; however, their limited brightness and energy efficiency, as well as their often unsatisfying viewing angle leave ample room for further improvements. The severe brightness and efficiency limitations of LCDs arise primarily because of the polarizers and absorbing color filters, that results in a large fraction of optical loss for the backlight.^50^ A simple illustration of the LCD components, along with an estimation of their light efficiency and brightness are provided in **Figure** **13**.

### 3.2 The Use of Quantum Dots in LCD Backlight Units

**Figure 14:**
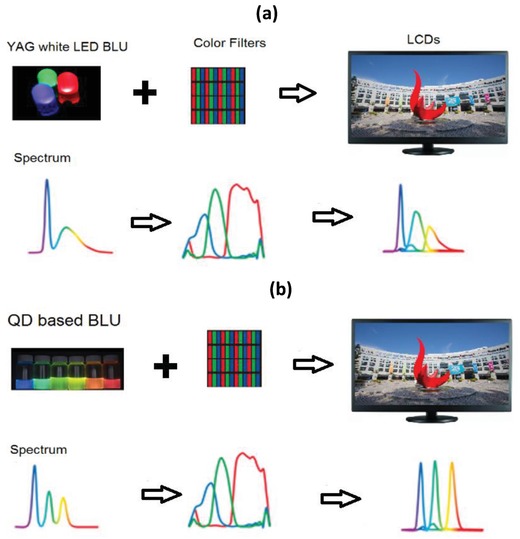
Comparison of LCDs equipped with different BLUs. a) White light from the YAG based LED BLU passes through the LCD panel and color filters to produce the full‐color image. b) The light from QD‐based BLU after passing through the LCD panel and color filters generates the full‐color images with highly saturated colors.

**Figure 15:**
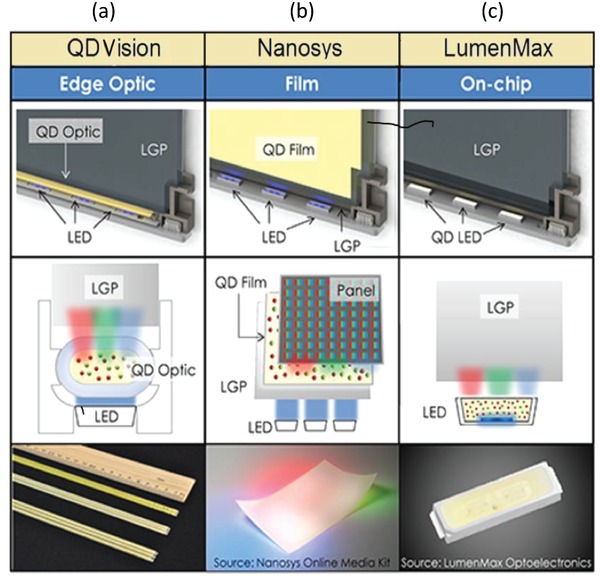
Contemporary QD‐based BLU configurations for the LCDs: a) QD Vision Inc. (Massachusetts), b) Nanosys (California), and c) LumenMax Optoelectronics (Taiwan). Reproduced with permission.^51^ Copyright 2015, Wiley.

A simple illustration and comparison of the color performance of two LCDs equipped with an Yttrium Aluminum Garnet (YAG) based white LED BLU and QD‐based BLU is shown in **Figure** **14**. With the spectral profile of the three well‐defined emission peaks, QD‐based BLU provides better color saturation and thus a wider color gamut. It has been reported that with an optimized QD‐based BLU, one can achieve a color triangle with greater than 115% of the NTSC standard in 1931 color space (via CIE coordinates).[qv: 2a,5] There are three possible configurations^51^ that have been explored for the QD‐based BLU for LCDs, as illustrated in **Figure** **15**: i) edge optics proposed by QD Vision (Massachusetts); ii) QD‐based enhancement films, abbreviated as “QDEF” and proposed by Nanosys (California); and iii) a QD‐on‐chip design proposed by LumenMax Optoelectronics (Taiwan).

QD Vision Inc. commercialized their QDs using an edge‐lit QD optic called Color IQ, which works with edge‐lit displays.^52^ In this configuration, the QDs are mixed with monomer were filled in an edge‐lit high precision glass tube and the monomer is polymerized. The tube acts as a strong barrier against moisture and oxygen, and is placed between the blue LEDs and a light guide in an edge‐lit backlight (Figure 15a). The concentrations of red and green converting QDs are adjusted to pass trichromatic white light, optimized for spectrally narrow color filters to maximize the throughput of the filters and deliver a wider color gamut than can be achieved with yellow phosphor LEDs. However, a significant portion of the light does not make it into the light guide, although some of it can be recaptured by integration of reflectors. Several companies adopted QD Vision's Color IQ system by 2016, including Hisense, Philips, Seiki, Sony, and TCL for TVs, as well as AOC and Philips for monitors. Importantly, the on‐edge approach for the QD implementation in displays requires a smaller quantity of QDs in comparison to the film approach. According to the estimates by Steckel et al., the worldwide consumption of QDs for the on‐edge approach, assuming 100% penetration of the QD in the LCD market, would be around 2–3 tons per year. In similar conditions, film type devices would require more than 200 tons per year.^53^

Nanosys also employed edge‐lit optics, particularly with films that generate trichromatic light with the right white color point, allowing a color filter to produce a wider color gamut than could be achieved with bichromatic light from a down‐converting yellow phosphor.^52^ They encapsulated the green and red emitting QDs together with small blue light‐scattering particles in the polymer film. The scattering particles were introduced in order to maximize the light usage. Nanosys' QDEFs can be used with either an edge‐lit or a direct‐lit BLU, which replaces the diffuser film by inserting red and green emitting QDs inside a film illuminated by blue LEDs (Figure 15b). Nanosys has so far been the biggest winner in the QD market, with their solution adopted in Samsung TVs ranging from 43″ to 88″ as well as other leading brands including Hisense, TCL, and Vizio.

Consequently, LumenMax came up with the idea of placing QDs directly on top of an LED chip, describing this structure as a QD‐LED (Figure 15c). This is a most desirable format because less QD material is required per display, and ease of system integration is maximized. However, this format demands QDs to withstand higher temperatures and excitation fluxes when compared with edge optic and film, and therefore, it requires further material and technological developments.

Zhonghuan Quantum Tech. together with researchers from the Southern University of Science and Technology in Shenzhen, China, proposed to use QD‐based luminescent microspheres (QLMS),^54^ as a type of a highly robust QD composite with long‐term operational stability. The fabrication procedure of their QLMS followed previously reported approaches,^55^ with several modifications. After soaking and swelling treatment, the average pore size of silica microspheres became enlarged and allowed the QDs solution to penetrate the pores and get access inside the silica matrix. Upon QD incorporation, the mesoporous silica structure prevented the QDs from aggregation, and reduced the contact surface between the QDs and the silicone, as well as between QDs and permeated oxygen and moisture promoting their stability. It was claimed that QLMS are fully compatible with current LED packaging processes and can be used as phosphors for direct on‐chip applications, meaning that tubes or films would be not required.

**Figure 16:**
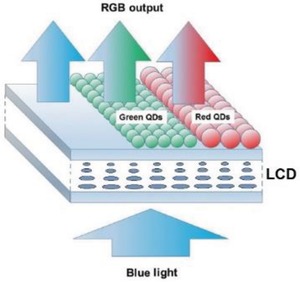
Scheme of a QD enhanced active color filter array. Reproduced with permission.[qv: 56a] Copyright 2017, IEEE Photonics Society.

Recently a QD enhanced active color filter array was proposed to replace conventional color filters. In this array, QDs are printed on top of the LCD panel and work as the down converting material.^56^ An illustration of this rather straightforward approach is given in **Figure** **16** where the blue light coming from the backlight is modulated by the LCD and later excites an array of QDs printed on the top.[qv: 56a] An advantage of this approach is that the LC device is only required to modulate a narrow bandwidth of the light, so that the thickness of the LC cell can be reduced greatly. This can greatly improve the LC response time and driving voltages. Furthermore, by replacing band pass color filters, which absorb a large portion of light, these emitting color filters can improve the efficiency of LCD devices if they have a high PL QY. Their placement in front of the display panel may also increase the color accuracy at higher viewing angles. At the same time, there are several issues with this approach, as it requires an additional optical filter to reduce backlight leakage and improve the contrast and viewing angle.[qv: 56b] Additionally, the LCD stack must be changed and the QD color filter needs to be placed after the analyzer, requiring some adjustments to the LCD production process.

### 3.3 The Use of Quantum Rods in LCDs

The conventional LCD structure is characterized by an overall optical efficiency of around 3–5% (Figure 13b), and the light loss across the color filters and polarizers is more than 70%. Field sequential color displays were introduced to manage the losses of color filters, but finding solutions to increase the polarization efficiency of modern LCD still imposes a challenge.^5^ The backlight equipped with polarized light emitters as a color conversion layer may increase the polarization efficiency of the polarizer films from 45% to 70%, and therefore, the overall efficiency of the conventional LCD may increase from 3–5% to 6–8% (Figure 13b).^39^

**Figure 17:**
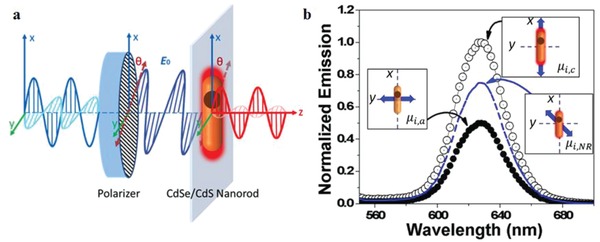
a) Anisotropic absorption and emission from a single core‐shell CdSe/CdS QR, with its long axis (*c*‐axis) parallel to the *x*‐axis in the xyz coordinates, b) Relative emission intensities of CdSe/CdS QRs aligned in *xy* plane at θ = 0°, 45°, and 90° with regard to the polarization azimuth of the impinging light. Reproduced with permission.^57^ Copyright 2018, Wiley‐VCH.

The polarized emission for the QR films depends on two factors: (i) the quality of the QRs, which is primarily defined by the florescent anisotropy, discussed above, and (ii) the quality of the unidirectional alignment of QRs, which is reflected by the order parameter. Additionally, because excitons in the QRs oscillate in mutually perpendicular directions, the QR alignment should not have any tilt for the alignment to leverage the full benefits of the polarized emission form QRs. Semiconductor QRs are characterized by the anisotropic absorption and emission, and therefore, by manipulating the angle of the polarization azimuth of the exciting beam in respect of the *c*‐axis of the aligned QRs, one can modulate the intensity of the emitted light, as illustrated in **Figure** **17**.^57^

In the composite films produced by the photoalignment technique, QRs are surrounded by LC molecules, which also show optical anisotropy and are situated orthogonal (in‐plane) to the QRs. The refractive indices and the relative permittivities of longer (*c*‐axis) and shorter axis (*a*‐axis) of LC molecules are *n*_∥_, *n*_⊥_, ε_∥_, and ε_⊥_, respectively, and the relative permittivity of the QRs is denoted as ε_rod_. Then, the local‐field factors of *c*‐axis and *a*‐axis can be expressed as:[qv: 17b,58]

(2) $$f_{\mathrm{LF},c}=\frac{\varepsilon_{⊥}}{\varepsilon_{⊥}+\left(\varepsilon_{\mathrm{rod}//}-\varepsilon_{⊥}\right)\alpha_{c}}$$

(3) $$f_{\mathrm{LF},a}=\frac{\varepsilon_{∥}}{\varepsilon_{∥}+\left(\varepsilon_{\mathrm{rod}⊥}-\varepsilon_{∥}\right)\alpha_{a}}$$

where the α_*a*_ and α_*c*_ are depolarization factors for *a*‐ and *c*‐axis, respectively.

The absorption *A* of a QR in the LCP matrix is related to the intrinsic absorption coefficient, *µ*_*i*_:

(4) $$\mu_{i}=\ln\left(10\right)\frac{A}{fL}$$

where *L* is the propagation distance of the impinging light, and *f* is the QR volume fraction in the film. Within the Maxwell Garnett model, the absorption coefficients of *c*‐ and *a*‐axis can be expressed in terms of the refractive indices and the dielectric functions of the surrounding molecules:^21^

(5) $$\mu_{i,c}=\frac{2\pi \;\varepsilon_{\mathrm{rod}}^{\prime \prime }{}^{}}{n_{⊥}\lambda }\;\;\;{\left|f_{\mathrm{LF},c}\right|}^{2}$$

(6) $$\mu_{i,a}=\frac{2\pi \;\varepsilon_{\mathrm{rod}}{}^{\prime \prime }}{n_{∥}\lambda }\;\;\;{\left|f_{\mathrm{LF},a}\right|}^{2}$$

Therefore, the absorption coefficient of the QR for the linearly polarized light will depend on the angle θ and can be derived as:

(7) $$\mu_{i,\mathrm{QR}}=\frac{2\pi \varepsilon^{\prime \prime }{}_{\mathrm{rod}}}{n_{⊥}\lambda }{\left|f_{\mathrm{LF},c}\right|}^{2}\cos^{2}\theta +\frac{2\pi \;\varepsilon^{\prime \prime }{}_{\mathrm{rod}}}{n_{∥}\lambda }{\left|f_{\mathrm{LF},a}\right|}^{2}\sin^{2}\theta$$

Thus, by controlling the polarization azimuth of the irradiating light, the emission intensity from aligned QR film can be well modulated.

**Figure 18:**
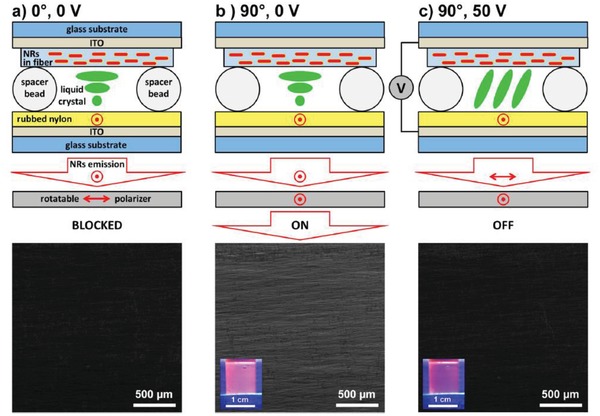
LC cell employing CdSe/CdS@SiO_2_@polymer nanofibers and corresponding fluorescence microscopy images depending on the polarizer orientation and electric field. a) Polarizer is parallel to the nanofibers (0°), and no electric field is applied. b) Polarizer is rotated perpendicularly to the nanofibers (90°), and no electric field is applied (ON‐position). c) Polarizer is maintained perpendicular to the nanofibers (90°), and a 50 V electric field is applied (OFF‐position). Insets of the fluorescence microscopy images in (b) and (c) show photographs of the LC cell using a portable UV lamp for the excitation. Reproduced with permission.[qv: 30b] Copyright 2015, American Chemical Society.

Aubert et al. synthesized aligned, silica coated CdSe/CdS QRs in polymer nanofibers by electrospinning.[qv: 30b] The uniaxial extensional flow experienced by the particles induced alignment of the QRs within the nanofibers. A subsequent parallel alignment of the nanofibers themselves resulted in a flexible film of 1.5 cm^2^, with a DOP of 0.45. It has been shown that such a self‐supporting film can induce the alignment of a nematic LC. By integrating them into a LC cell with a polarizer, the simple liquid crystal device constitutes an efficient way of electrically switching on and off polarized light by 5.0 volts and fully preserving the polarization ratio over a large area (**Figure** **18**).

**Figure 19:**
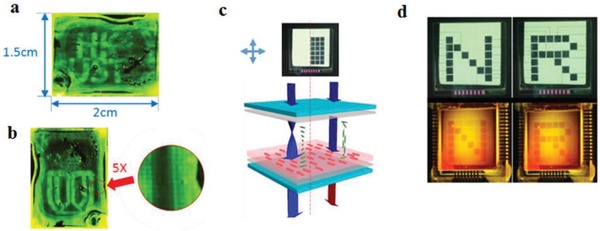
Strategies to generate emission contrast from photoaligned QR films. a,b) Two images are projected on one unidirectional aligned QRs substrate by polarized backlight through a VAN LCD, which facilitates to control 16 polarization azimuthal angles of individual pixels (200 µm × 200 µm). The inset image in (b) presents an array of pixels with 5 × magnification. c) In‐LC cell optically addressable emissive QR display. d) The patterns “N” and “R” with backlight under cross polarizers (upper part), and polarizer‐free emission patterns “N” and “R” with impinging light (λ = 450 nm) and 480 nm cut‐off color filter (bottom part). Reproduced with permission.^57^ Copyright 2018, Wiley‐VCH.

Zhang et al. presented a fixed information display, where the image is defined by the pre‐aligned QR domains in the film, and the contrast can be controlled with a LC based polarization rotator (**Figure** **19**).^57^ It was demonstrated that using a VAN LCD cell driven by an array of thin film transistors, an optically addressable random information display which is able to show any image with a substantial depth of gray scale could be produced. The LC cell with QRs showed the same properties as a regular LC cell under crossed polarizers but added controllable down conversion emission from aligned QRs (Figure 19). Because of the in‐cell structure, such optically addressable devices have large potential for efficient and stable color conversion in photo‐emissive displays,[qv: 30b,59] security applications and other applications related to displays and photonics.

### 3.4 The Color Performance Optimization for the Backlights with Quantum Dots and Rods

**Figure 20:**
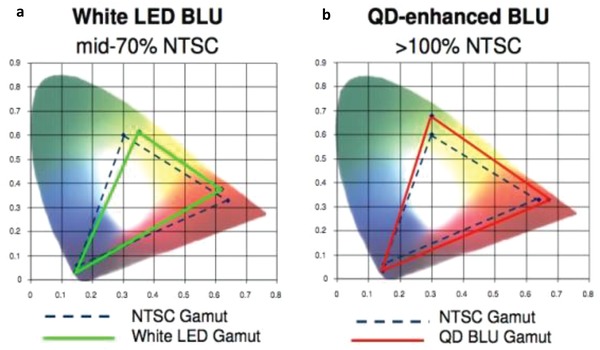
Comparison of the color performance of two LCDs equipped with a) white LEDs and b) QD backlight unit are compared to NTSC standard for television. (Image courtesy: QD Vision).

To date, QDs have been heavily explored for LCDs with enhanced optical performances. In this respect, QD Vision, Inc. has compared the color performance and power efficiency for two LCDs equipped with white LED backlight and QD backlights, as shown in **Figure** **20**. The area within the CIE color triangle for the LCD display with a QD backlight is larger than the 100% of the NTSC in 1931 color space.[qv: 2a,56a] Furthermore, they claimed that the PL bandwidth should be smaller than 30 nm for a wider color gamut and better optical efficiency. In addition to the saturated color, the QD‐based backlight also offers better optical efficiency; it was estimated that QD equipped LCDs can save at least 20% more power than conventional alternatives.^60^ QD Vision has also proposed a new standard called “Color Nits” for measuring brightness and luminance in the latest high‐performance displays that support high dynamic range and wide color gamut. The “Color Nits” metric is derived from a formula that takes into account varying spectral profiles and the subtle differences between perceived brightness and actual luminance. This concept integrated some aspects of the so‐called Helmholtz–Kohlrausch effect, which states that more saturated colors can appear brighter than less saturated ones of equivalent luminance, meaning that wider color gamut results in higher perceived brightness levels.^61^

**Figure 21:**
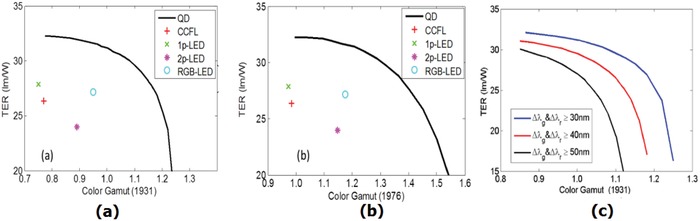
Color gamut optimization for the QD LCD in a) 1931 color space and b) 1976 color space. c) The Pareto front for the QD backlight for QDs with different PL FWHM. Reproduced with permission.[qv: 62b] Copyright 2013, The Optical Society.

Wu and co‐workers studied the effect of the PL peak position and its FWHM on the optical performance of an LCD equipped with QDs.^62^ There are two characterization parameters for QD‐based LCDs, the color gamut and the total efficiency of the radiation (TER). Both of these parameters were evaluated in ref. [qv: 62b] using a function of six different parameters of the QDs (e.g., the PL wavelength (*λ_i_*) and FWHM), where *i* is r (red), g (green) and b (blue), and human eye sensitivity. The two proposed equations are:

(8) $$\mathrm{Color}\;\mathrm{gamut}=F_{1}\left(\lambda_{b},\Delta \lambda_{b},\lambda_{g},\Delta \lambda_{g},\lambda_{r},\Delta \lambda_{r}\right)$$

(9) $$\mathrm{TER}=F_{2}\left(\lambda_{b},\Delta \lambda_{b},\lambda_{g},\Delta \lambda_{g},\lambda_{r},\Delta \lambda_{r}\right)$$

where λ is the wavelength and Δλ is the FWHM for each color, r, g, and b. Since color gamut in the above equation can be defined either in CIE 1931 or CIE 1976 color space, they have performed two separate optimizations, as shown in **Figure** **21**. The performance of the conventional backlight sources is also included in the same figure for comparison. In Figure 21a, the black curve represents the so‐called “Pareto front” of QD backlight performance. The QD backlight could vary from low color gamut (77% NTSC) but high TER (32.3 lm W^−1^) to high color gamut (123% NTSC) but low TER (<20 lm W^−1^). The trade‐off between TER and color gamut is obvious because the gain of one metric results from the loss of the other.[qv: 62b] The QD backlight outperforms conventional backlight sources in both system: it can reach a color gamut of 115% of the NTSC standard in CIE1931 color space and 140% of the NTSC standard in CIE 1976 color space, while maintaining the same energy efficiency as conventional light sources. The effect of PL FWHM on the two parameters is shown in Figure 21c. Later on, the same group optimized the optical efficiency and color gamut simultaneously to realize Rec. 2020 for QD LCDs;[qv: 62a] 97% of the Rec. 2020 color gamut could be achieved while maintaining a reasonably high optical efficiency for both fringe‐field‐switching and multi‐domain vertical‐alignment LCDs.

## 4 Conclusions and Outlook

The Cd‐based light‐emitting nanocrystals have been thoroughly explored, and the current state of art for the synthesis of these materials allow us to achieve PL QY ≈100% and FWHM of the emission ≈30 nm, both highly suitable for display applications. However, because of the limitations on Cd toxicity, several markets have imposed severe limitations on the use of these materials in consumer products. Currently, the concentration of Cd in displays is restricted to 0.2 µg mm^−2^ by the *EU RoHS2* directive. We note that the Cd concentration for the existing QREF is ≈0.05 µg mm^−2^, which is already below the limit stated in the *EU RoHS2* directive. However, it would be highly desirable to avoid the cadmium completely or find a way to reduce the concentration of Cd well below the limit to avoid any potential risk for humans and environment. On the other hand, stability of the Cd‐based nanocrystals in an open environment is not the main issue for QDEF and QREFs, because mostly they are well encapsulated in the polymer films and their stability is thus greatly enhanced.

For cadmium‐free QRs, indium phosphide can be a potential replacement candidate, but the bandgap for bulk InP is 1.35 eV, smaller than that of CdSe. In order to reach the same emission wavelength, the core size of the InP QDs must also be smaller than that of CdSe. Smaller bandgaps and smaller particle sizes lead to much stronger confinement effects, and as a result, the emission spectrum of InP QDs is more susceptible to particle size variations, and its FWHM is somewhat broader (>40 nm). This corresponds to a 70−80% Rec. 2020 color gamut, depending on the color filters employed. However, backlights based on InP QDs have already been widely used in TV products, such as in Samsung QD TV series, and have exhibited excellent performance and long lifetime.^63^ InP QDs are non‐toxic, however, there are still synthetic challenges, given that phosphide precursors are highly reactive and potentially dangerous. This issue is even worse for anisotropic NCs based on InP, given the synthetic demands of higher concentration and, often, more reactive precursors. One approach proposed by Nanosys is a “greener” QD system obtained by combining the Cd‐free and low Cd‐based QDs.^64^ This hybrid approach not only retains 90% Rec. 2020 color gamut but also complies with the RoHS regulation.

Another approach to the toxicity issue could be to use cesium lead halide perovskite QRs as the polarized emitter. These materials are very similar in synthesis and material properties to II‐VI and III‐V nanocrystals and display high QY and small FWHM of emission, making them well suited for LED applications. Several recent breakthroughs with these materials have occurred, and the field has experienced unprecedented development in just a few years' time.^65^, ^66^, ^67^ While the current stability limitations and the presence of toxic Pb may again be a limiting factor for commercialization,^68^ recent results of high efficiency electroluminescent perovskite LEDs have generated significant interest in CsPbBr_3_ as a down‐conversion emitter.^65^, ^66^, ^67^ Nanocrystals of metal lead halides have been demonstrated with multiple routes to synthesis, including in situ and in polar solvents,^68^, ^69^ and color tunability, including halide exchange and quantum size effects_,_ to achieve linewidths as thin as 17 nm in FWHM. Emission from perovskite QRs has been found to display strong polarization anisotropy.^68^ Perovskite nanowires synthesized via A‐group substitution suggests that a large family of anisotropic, inorganic perovskite nanocrystals with varying properties may be synthesized via careful selection of the reaction precursors.^70^ Finally, in situ perovskite nanorod synthesis and alignment,^71^ and early stage LCD pixels have been reported, albeit with limited performance thus far. All this suggests that several innovative approaches are possible to achieving polarized emission from perovskites in LCDs.

The polarized emission from well‐aligned semiconductor QRs has a great potential to improve the efficiency of modern LCDs, and there have been several approaches demonstrated in literature to realize such an alignment, as summarized in Table 1. While DOPs of resulting systems may not be sufficient to completely eliminate the need for the polarizers, they can reduce losses in the LC polarizer considerably. Photoaligned QREFs employing semiconductor QRs with polarized emission for brightness enhancement of the BLUs have been demonstrated, and these showed improved light efficiency in addition to color enhancement for LCDs.[qv: 35c,39] Additionally, the dipolar emission of the QRs offers higher output light coupling in comparison to QDs. For the LCD backlight application, there are two scaling factors for the QREF brightness: i) the PLQY of the QR and ii) the DOP of the aligned QREF. Thus, for the real benefits it is important to achieve both high PL QY and high DOP in the QREFs. Figure 13b shows that the brightness of the LCD can be increased from 4.8% to 7.9%. The narrower emission (FWHM < 30 nm) and shorter emission wavelength for the green emitters (PL maximum ≈520 nm) would further help to enhance the color performance of the QREF. This can be achieved by both optimization of the traditional QR synthesis or by employment of alternative nanomaterials with polarized emission such as II‐VI semiconductor nanoplatelets, or perovskite nanocrystals.

## Conflict of Interest

The authors declare no conflict of interest.
